# The Compendium of U.S. jails: creating and conducting research with the first comprehensive contact database of U.S. jails

**DOI:** 10.1186/s40352-021-00137-7

**Published:** 2021-05-19

**Authors:** Chelsea M. A. Foudray, Camille Kramer, Danielle S. Rudes, Carolyn Sufrin, Eliza Burr, Trisha Parayil

**Affiliations:** 1 Department of Criminology, Law and Society, George Mason University, 4400 University Dr, Fairfax, VA 22030 USA; 2 Department of Gynecology and Obstetrics, Johns Hopkins School of Medicine, Baltimore, MD USA; 3 Department of Criminology, Law and Society, Center for Advancing Correctional Excellence (ACE!), George Mason University, Fairfax, VA USA; 4 Krieger School of Arts & Sciences, Johns Hopkins University, Baltimore, MD USA

**Keywords:** U.S. jails, National Jails Database, National Jails Compendium, Pregnancy, Opioid use disorder, Medications for opioid use disorder (MOUD)

## Abstract

**Background:**

Millions of people pass through U.S. jails annually. Conducting research about these public institutions is critical to understanding on-the-ground policies and practices, especially health care services, affecting millions of people. However, there is no existing database of the number, location, or contact information of jails. We created the *National Jails Compendium* to address this gap. In this paper, we detail our comprehensive methodology for identifying jail locations and contact information. We then describe the first research project to use the *Compendium*, a survey assessing jails’ treatment practices for incarcerated pregnant people with opioid use disorder.

**Results:**

This study sent surveys electronically or in paper form to all 2986 jails in the *Compendium,* with 1139 surveys returned. We outline the process for using the *Compendium,* highlighting challenges in reaching contacts through case examples, cataloging responses and non-responses, and defining what counts as a jail.

**Conclusion:**

We aim to provide tools for future researchers to use the *Compendium* as well as a pathway for keeping it current. The *Compendium* provides transparency that aids in understanding jail policies and practices. Such information may help devise interventions to ensure humane, evidence-based treatment of incarcerated people.

## Introduction

In 2018, there were an estimated 10.7 million jail admissions, with nearly 740,000 confined in jails and thousands returning to communities from jails daily (Zeng, 2020). Jail time impacts those incarcerated and their families, employers, and communities, and disproportionately affects people of color and other marginalized groups. Despite this broad-reaching influence and that jails are publicly funded institutions, we cannot definitively confirm the number of jails in the U.S. Without systematic information about where and how many jails exist, the implementation of standardized, safe health care services within jails remains challenging. In contrast, prisons have easily accessible, centralized accountability systems including websites and addresses for state departments of corrections and the Federal Bureau of Prisons. There is no available database listing the location and existence of U.S. jails.

Since 1982, the Bureau of Justice Statistics (BJS), a branch of the U.S. Department of Justice, has collected demographic data about people in jails; the most recent 2013 Census of Jails estimated there were 3134 jails (Bureau of Justice Statistics, n.d.; Minton, Ginder, Brumbaugh, Smiley-McDonald, & Rohloff, 2015). While BJS makes all of its data, collection tools, and methodology public, the studies are limited in scope (Chari, Simon, DeFrances, & Maruschak, 2016; Maruschak, 2016). Contact information for these jails is not publicly available, nor is the means through which BJS confirms that the institutions are, in fact, jails. BJS defines jails as “correctional facilities that confine persons before or after adjudication and are usually operated by local law enforcement authorities,” (Minton et al., 2015, p.20) in addition to confining people for more than 72 h (Bureau of Justice Statistics, 2018). Still, there are caveats to these definitions, including federally operated jails and single unified prison/jail systems.

Data from jails is important because jails are ground zero for numerous justice and equity concerns, including issues of policing, health, poverty, and racism. To address the gap in data and facilitate research about U.S. jails, the National Institute of Corrections (NIC) partnered with researchers at George Mason University to compile and validate a *National Jails Compendium*. In this paper, we outline the methodology for assembling the *Compendium*—the largest, most comprehensive database of jails to date. We then describe the first use of this database for a national, cross-sectional survey collecting data on the availability of medication treatment for opioid use disorder (MOUD) for pregnant people in jails. Our aim is to highlight unique methodological insights for conducting a large-scale study of all known US jails and highlighting the nuances of studying our nation’s disjointed and highly localized jail system. We outline lessons learned from recruitment processes, categorizing survey responses and non-responses, and other challenges through case examples. This account will aid future researchers and users of the *Compendium*.

## Methods: creating a U.S. jails database from scratch

### Collaboration and research context

Leaders at NIC (a federal agency that provides technical assistance and guidance for policymakers and correctional institutions) and the Bureau of Prisons (BOP) recognized both the lack of a comprehensive contact database of jails in the U.S. and the value of having such a list for research and policy. NIC and the BOP therefore contracted with Dr. Danielle Rudes' and Chelsea Foudray's research team at George Mason University in 2019 to assemble a *National Jails Compendium,* a database with verified contact information for every U.S. jail facility.

Simultaneously, The Advocacy and Research on Reproductive Wellness of Incarcerated People (ARRWIP) research team at Johns Hopkins University School of Medicine met with leaders in the jails division of NIC and the National Sheriffs Association (NSA)—a non-governmental, professional association for local sheriffs with state-level affiliates—to discuss the importance of jails providing appropriate treatment to pregnant people with opioid use disorder (OUD) housed in their facilities and ARRWIP’s plans to conduct a survey on jail’s practices around OUD in pregnancy. Given NIC’s recognition of the significance of prioritizing treatment for pregnant people in jails, a plan emerged to administer this survey using the *Compendium*. Prior to survey distribution, the GMU and ARRWIP teams discussed the *Compendium*, its intended use for the survey, and brainstormed strategies for distribution and anticipated pitfalls.

Building the *Compendium* required an extensive, comprehensive effort to identify, catalogue, and collect information on all U.S. jails (non-tribal, adult, non-private). To provide transparency and help future researchers understand the methods involved in the *Compendium’s* creation, this section outlines inclusion guidelines, search strategies, and data management of the three-phased approach to locating and verifying jails’ contact information.

### Inclusion and exclusion criteria

We crafted clear inclusion and exclusion criteria for what counts as a jail. Our working definition included any carceral institution (non-prison) that goes beyond having just holding cells. Jails listed in the *Compendium* are facilities that hold detainees pre- and post-adjudication. Using these criteria, we identified jails for U.S. adult populations that are non-tribal and non-private in cities or counties in all 50 states plus Washington, DC. To ensure a comprehensive *Compendium*, facility size did not matter.

When a jail accommodated different populations in two or more separate buildings (i.e., adults/juveniles; males/females), we listed each facility separately even when the facilities had the same physical address, but only included the adult units. The team made this decision because separate buildings/units may operate differently. However, when two different groups were housed in the same building, the facility was treated as one facility, as staff in these units are often fluid. We excluded facilities that only operate as reentry/work release facilities, weekend programs, or serve as temporary holding units (i.e., 48-h holding cells). Only active and presently open facilities were included. These criteria, along with decisions as to which information to include in the *Compendium*, were established in discussion with the NIC and the BOP.

### Search strategy

The team searched the internet using Google to find jails in each U.S. state and D.C. Phase 1 of the search entailed compiling information provided by a few research teams at universities and non-profit organizations. In Phase 2, we expanded the search to identify counties in each state for a more targeted search, using terms such as *[state] jails, [county] jails*. Additional verification included searching for sheriff’s offices related to each jail. From there, we followed the website and/or other available information to verify and locate jails. For comprehensiveness, the team searched for each facility’s physical jail address, phone number, email, contact person—sheriff, police chief, other jail administrator—and whenever possible, the number of beds. Initial searches yielded few addresses and phone numbers, and little regarding jail leaders’ names and email addresses.

To facilitate searching, we used information from Bureau of Prisons (BOP)/NIC, utilizing connections with research teams involved in jail-based research as well as available websites/lists. Since Phase 2 demanded exhaustivity, we split the initial search between two team members, each searching 25 states to create a fuller list. This phase yielded approximately 80% of the included jails. The main sources of information included state Sheriffs’ Associations and “offender locator” sites. We also used informational websites, including PrisonPro, SearchQuarry, Public Records, and InmateAid.

During Phase 3, the team creatively approached missing jail information. We searched for personnel affiliated with jails and information about individual jails. We did this by searching county/city Facebook pages, Twitter accounts, LinkedIn, Sheriff’s Associations, local news postings from particular jurisdictions, and other avenues available. While this information was often negligible, several times we located key information about a jail via these untraditional methods. We also searched using Google Maps and made direct contact with facilities through website submission forms or phone calls. When these methods failed to provide the necessary information, we identified the sheriff or police chief as the main point of contact.

As part of this phase, we also enlisted the help of nearly 60 well-trained undergraduate research assistants to do a fourth-round search for any missing jails that conformed to inclusivity requirements. These undergraduate assistants were thoroughly briefed on the purpose of the *Compendium*, trained in the search strategy employed by the team, trained in how to speak with jail administration, and closely supervised by the lead researchers. We assumed that jails were missing from our dataset due to the lower number of jails found using the aforementioned search techniques, as compared with BJS’s estimate of the number of jails in the U.S. (*n* = 3134) (Bureau of Justice Statistics, 2018). The research assistants verified existing information and identified missed jails. We found approximately 20% of the jails in the *Compendium* during this search. Any additional facilities found during this phase were verified by the lead researchers. The research assistants followed-up on any inconsistent information by contacting the facilities (generally via telephone), though facilities were often unable or unwilling to provide necessary information. More than 80% of jails contacted hung up. The inability to establish direct contact with facilities also meant we were unable to expand upon the *Compendium* through snowball sampling. When that occurred, we searched for that facility again on all of the websites we utilized in Phases 1 and 2, pinpointed several sources, and included the most recent information.

### Data management

To build the *Compendium*, the team created an Excel file containing a single tab for each of the 50 states and for D.C. that still operates as a living document and is continually updated. Each worksheet in the file contains rows for individual jails and columns to capture jail name, physical address, phone number, contact person, email address, and two “optional” columns for number of beds and miscellaneous information (Table 1).

**Table 1:** Overview of Data Capture

Data Capture Categories	Definitions of Captured Data
**Jails**	• Located in **U.S.** (any state or DC), non-Tribal, non-private • Hold **adult** residents^a^ (male, female or both). Can also hold juveniles, but not *only* juveniles • Hold residents **post-adjudication/trial**, but can also hold **pre-trial,** temporary and reentry residents • No single-cell/bed or couple-cell/bed holding units only (i.e., drunk tanks) without any other jail-criteria
**Addresses**	**Actual jail address**, not the mailing address (and not the address to send resident mail); Jail/facility address, not the sheriff’s/police chief’s office address, if they are in separate buildings/locales
**Phone Numbers**	**Main jail phone line**; Not the number to call to talk to a resident, not the Sherriff’s main line and not any person’s individual line (as people will change positions/phones)
**Contact Person(s)**	**Any contact** for Sheriff, jail, or any jail person; When nothing jail-specific is available, include local Sheriff/Police Dept.
**Email Addresses**	**Person in charge of or running the jail**; Prefer Captain, Deputy, Warden, Lieutenant; If not, then list Sheriff’s or Chief of Police

^a^In keeping with the growing and important person-first language tradition, we opt to use the term “residents” to refer to individuals confined in U.S. jails. We choose this term because it denotes a particular locale rather than acting as a label or judgement on an individual the way words like “inmates,” “prisoners,” and “convicts” do. We could have used “individuals who are incarcerated”—in true person-first style. However, this language is in passive voice and adds many more words to the manuscript. Thus, we opted for the term residents. Whenever other scholars, residents, or staff use the terms “inmate” or “prisoners,” however, we include that term as it is an in vivo representation of the language used by prior researchers and our prisons, units, and the individuals within them

For quality control, the research team partnered with the Vera Institute for contact data verification during the late spring of 2019, as Vera was collecting information on U.S. jails that coincided with the *Compendium* creation. This cross-check resulted in three jails added to the *Compendium*.

As of June 2020, the *National Jails Compendium* consists of 2953 facilities. This number is nearly 200 jails lower than the 2013 BJS estimate (Bureau of Justice Statistics, 2018). This lower number may partially be due to jail closures and the difficulty of locating and identifying U.S. jails. It must be noted, however, that the BJS utilizes broader inclusion criteria than the *Compendium*. The BJS includes special jails such as release centers, halfway houses, and work farms^6^, while the *Compendium* excludes such facilities. Additionally, compared to jail documents previously used for jail-based research, such as the Annual Survey of Jails, the *Compendium* used an exhaustive strategy to collect facility-level information about jails rather than a representative sample to determine characteristics of residents within jails. As such, the *Compendium* provides a wider opportunity for research within jails.

## Results: first use of the *National Compendium* to research jails

### Processing the compendium for study recruitment

The GMU team shared the *Compendium* with ARRWIP in July 2019, at which point there were 2986 jails in the database. The research study was approved by the Johns Hopkins School of Medicine’s Institutional Review Board.

We initially planned to administer surveys solely online using the web-based, secure Research Electronic Data Capture (REDCap) platform (Harris et al., 2009). However, nearly one-third of jails (*n* = 921) lacked an email address. Thus, we divided the *Compendium* into two groups: Wave 1 consisted of electronic survey administration via REDCap to jails with an email address; Wave 2 involved mailing surveys via the U.S. Postal Service for jails with no email address and Wave 1 jails that did not respond after two email reminders. Each method yielded valuable information and illuminated the nuances of large-scale jail-based research.

### Wave 1: electronic recruitment and survey administration

From the *Compendium*, we created and cleaned a new Excel file, containing only jails with an email address, and imported into REDCap. Cleaning consisted of consolidating jail entries with two contact persons and email addresses listed. If there were two contact persons for the same facility, we selected the first one listed or the more professional of the two based on username and email server (e.g., Gmail, Hotmail.). We addressed recruitment emails to sites without a contact person as ‘Dear Sheriff.’ For survey purposes, we consolidated entries where contact information for multiple facilities was the same (*n* = 59), although the *Compendium* guidelines defined different units at the same jail as different entities. We did this because, presumably, they were under the same operational oversight and would therefore have similar health care policies across facilities, to reduce duplicate responses, and to limit recruitment burden. Wave 1 included a total of 2006 contacts with email addresses from the original *Compendium*. We added an additional 11 contacts from NIC’s Large Jail Network and NSA Executive State Heads who were not in the initial contact database, bringing the total to 2017 contacts with email addresses.

Electronic recruitment consisted of an initial email invitation and survey link, followed by reminders 2 and 4 weeks later. To maximize chances that people would open the emails and signal this was about jails and opioids, we created a dedicated email address for the study, opiates.jailmoms@jhu.edu. We used the word “inmate” in our communications with potential subjects to use language that would resonate with their daily operations and increase their chances of participating; however, we recognize the importance overall of using person-centered language. Our study was backed by NSA, and we leveraged this support by including, with permission, their logo and endorsement in our recruitment email. As an incentive, the research team also pledged, for each completed survey, a donation to one of two charities of the jail’s choice: First Responders Children’s Foundation or Phoenix House. This donation was the same amount for all facilities. We chose this instead of direct compensation because we anticipated many respondents would be unable to accept individual remuneration.

Three weeks after sending the mass initial survey invitation—and after at least one reminder—a contact at the NSA sent an email to all state executive directors endorsing the survey and urging them to encourage member jails to participate. We sent a final reminder email to non-respondents 3 months after Wave 1 began. If we did not receive a response 3 weeks after this, they were counted as non-respondents.

Despite these efforts, the Wave 1 response rate was approximately 10%, with a significant amount of undeliverable recruitment emails (*n* = 257). The failed delivery could be attributable to changes in jail leadership, incorrect email addresses or server rejections, despite the validation process used in creating the *Compendium*.

### Wave 2: paper recruitment and survey administration

Wave 2, paper recruitment, was more complex and required more planning, time, and person-power. Initially, we designated Wave 2 for the 908 contacts without email addresses. Due to the low Wave 1 response rate, we decided to add all 1807 Wave 1 non-respondents to Wave 2, making the total mailing list roughly 2715 contacts.

**Fig. 1:**
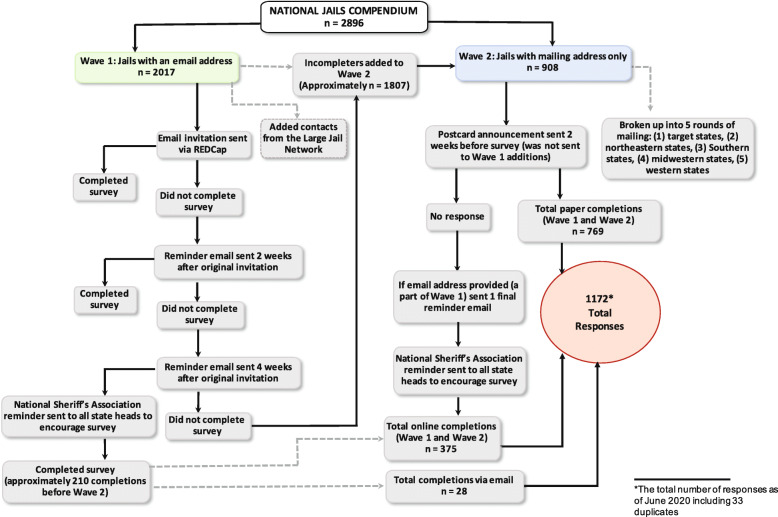
National Jail Survey Recruitment Flowchart

We organized Wave 2 mailings in a staggered fashion to states based on geographic region to manage the large number of contacts. To maximize responses, we sent a postcard to alert the jail to the survey’s impending arrival. To lend legitimacy to the mailing, the postcard displayed both our institution’s and the NSA logo, and featured an open link to the survey so recipients could access and complete the survey online. This system allowed us to efficiently send out all materials within 2 months. If an individual’s name was not listed in *The Compendium*, we addressed surveys and postcards to the jail’s “Health Services Administrator.” Each mailed survey included a pre-addressed, stamped return envelope to return the completed survey and an additional ten pages for sharing pertinent jail protocols, if applicable; people could also return the survey by scanning and emailing it. (Fig. 1, Table 2).

**Table 2:** National Jail Survey Recruitment Method and Survey Yield

Study Team Action	Number Participants Targeted	Number of Responses Received
Wave 1 Electronic Recruitment (before Wave 2)	2017	210
Added Contacts from Large Jail Network	32^a^	11
Wave 2 Paper Recruitment	908	254
Added non-responders from Wave 1 to Wave 2	~ 1807	638
NSA endorsement through state head emails^b^	–	26
**TOTAL RESPONSES**		1139

^a^Some facilities from the Large Jail Network were in the *Compendium* but the LJN listed a different contact person in which we included (of the 32 contacts, 11 new facilities were added to the *Compendium*)

^b^We suspected those who completed the survey who were not a part of our direct recruitment methods learned about the survey through the National Sheriff Association’s endorsement through state head emails

### Managing responses

Paper recruitment yielded far more completions and correspondence. Some paper surveys had inconsistencies that demonstrate overarching themes specific to jail research, such as leadership turnover, delivery to an inappropriate person, jail leadership miscommunication and possibly misunderstanding of health care services, and difficulty pinpointing the facility’s physical address, including reports of “no jail” at the specified location. However, most returned surveys were completed without issues.

We received a total of *n* = 1139 returned surveys (39% response rate) to the national jail survey between September 2019 and June 2020 (with most completed by December 2019): 375 via REDCap, 769 mailed responses and 28 emailed responses. Of the 375 online completions, 32 respondents were solely in Wave 2, meaning they followed the link on the recruitment postcard to complete the survey online. These responses encompassed all correspondence received from recipients including: (1) completed and partially completed surveys (*n* = 853); (2) not applicable responses (*n* = 226); (3) duplicate responses from the same facility (*n* = 33); (4) ‘already completed’ responses despite incompletion (*n* = 9), and (5) ‘addressee not here’ responses (*n* = 18). At our invitation, 74 jails submitted (9 via REDCap, 69 via USPS) written jail policies and protocols pertaining to MOUD treatment for pregnant people.

Twenty respondents incorrectly reported that they or someone else at their jail had previously completed the survey. We sent these individuals a personalized letter and requested completion, which yielded an additional 11 surveys.

**Fig. 2:**
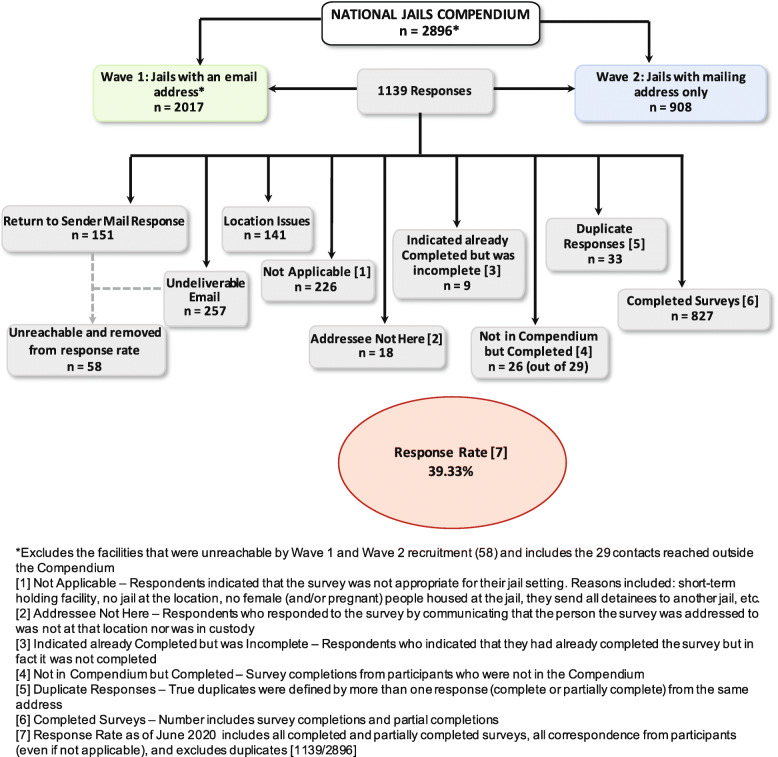
National Jail Survey Responses

We created an option for snowball sampling, asking recruited people to share the survey link with others, which yielded 26 additional responses (included in the total responses and response rate) from jails that were not a part of our version of the *Compendium*. We suspect they may have received the link from colleagues or through the NSA state emails. These 26 additional facilities were added to the *Compendium*. Figure 2 illustrates the categorization of all responses received from the National Jail Survey.

### Challenges & case examples

This section describes useful lessons from this large-scale jail health services survey. We highlight major challenges encountered, how challenges were handled, and provide representative case examples. This information informs the practicality using the *Compendium* for future research studies, highlights research limitations within local institutions of detention, and begins conversations about overcoming such barriers (Table 3).

**Table 3:** Challenges & Case Examples

**Undeliverable** **Surveys**	
***Definition:*** *Surveys that were not delivered to the recipient as intended either because of a failed email delivery (Wave 1) and/or a mailed survey coming back as ‘return to sender’ (Wave2).*	
Wave 1: Delivery Failed Messaging	
(1) The email was rejected by recipient email system	
(2) The recipient mail server may be temporarily offline or temporarily unable to accept messages	
(3) A problem occurred while delivering the message to the recipient	
Wave 2: Return to Sender, Unable to Forward Messaging	
(1) Not deliverable as addressed	
(2) Insufficient address	
(3) No mail receptacle	
(4) No such number	
(5) Attempted—not known	
During Wave 1, our study team received a significant number (*n* = 257) of undeliverable recruitment emails sent via REDCap. Based on the delivery failed messaging, the study team attributed the undelivered emails to recipients’ servers blocking emails from an unknown email address that may be suspected as spam or alternatively, attributable to incorrect or outdated email addresses from the *Compendium*. However, those who did not receive the email in Wave 1 were recruited again in Wave 2. In Wave 2, we received a large number (*n* = 151) of ‘return to sender’ returned envelopes due to incorrect addresses. If a recipient had an undeliverable email and a ‘return to sender’ mailing response in Wave 2, we designated those contacts as unreachable (*n* = 14) by both recruitment methods. Recipients solely in Wave 2 who had a ‘return to sender’ response (*n* = 44) were also deemed unreachable. We attributed Wave 2 return to sender responses to incorrect physical mailing addresses. Unreachable contacts were removed from the response rate.	
**We acknowledge that leadership positions in jails are often times elected officials which could contribute to outdated contact information. However, inaccurate physical addresses lend to the notion that general information on jails is limited to the public who they purposely serve, a theme also highlighted in the creation of the** ***Compendium*. The undelivered surveys ultimately help us and others understand the difficulties with getting in contact with jails for research (and/or other purposes) and suggests that the existence of a** ***Compendium*** **would need to be updated regularly.**	
**Addressee** **Not** **Here**	
***Definition:*** *Surveys that were received and returned [but not completed] indicating that the person the survey was addressed to was not at that physical location.*	
The study team addressed all mailed surveys in Wave 2 to the listed contact person provided in the *Compendium*; as noted, ‘Health Services Administrator’ was used if no contact person was listed. Unpredictably, some unopened surveys (*n* = 27) were returned with messaging indicating that the recipient was no longer there nor in custody.	
Addressee not here Messaging:	
(1) Addressee released (no forwarding address)	
(2) Not at address	
(3) Inmate no longer at [facility]	
(4) No inmate [with] this spelling	
(5) Inmate unknown	
(6) Not in custody	
In response, the study team resent all surveys to those facilities in new envelopes and addressed them to the attention of the ‘Health Services Administrator.’ Upon the second attempt, nine of the 27 completed the survey. The remaining 18 were also included in the overall response rate (but will excluded from data analysis) since the respondents returned the survey with a note.	
**The study team did not send any surveys to incarcerated people, however, whoever handled the mail did not know the contact person listed in the** ***Compendium*** **and assumed it was for a detainee. This, again, suggests there is either inaccurate reporting of who works in the facility or high turnover.**	
**True** **Duplicate** **Responses**	
***Definition:*** *When respondents submitted more than 1 survey response from the same physical location according to the Compendium; surveys could have been completed by the same person or multiple people.*	
The study team and representatives from the Johns Hopkins University BEAD (Biostatistics, Epidemiology and Data Management) Core discussed the many complexities of the types of survey responses we received and decided to use the basis of reporting from the same physical location as the primary criterion for a ‘true duplicate.’ This helped us eliminate instances where there was geographical reporting overlap with neighboring counties, cities and regional facilities (further discussed in the ‘Location Challenges’ section).	
We received duplicate responses (n = 33)* from the same facility despite a screener question in place to prevent duplicates demonstrating instances of miscommunication in jail settings. Duplicates included both fully completed and partially completed surveys. Some duplicate responses were submitted by the same person; in other instances, two jail leaders (e.g. the Warden and Captain) both submitted a response. In all but three instances, the respondents were custody personnel, not facility health care providers, which could contribute to the misinformation. The amount of discrepant information varied by facility and these facilities were not unique based on any characteristics we are aware of (i.e. geography, size, etc.). We preliminarily reviewed duplicate submissions to determine consistency in answers to help inform our resolution approach. Our initial review showed discrepancy in the reported data warranting us to contact the respondents to get an accurate depiction of that facility’s response. Only 2 of the 33 duplicates submitted the same information on both reports.	
**Even though duplicate reporting was discouraged it yielded important insights about how jails operate and how they interpret and report their health care services [specifically for pregnant people with OUD]. In some cases, a duplicate report by the same person exhibited different responses to the same questions. In other instances, the two leaders reported fundamentally different responses, even about who they detain at the facility. Duplicates further emphasize the incongruences of the inner-workings of jail facilities.** *The duplicates were only counted once in the sum of total responses	
**Not** **Applicable**	
***Definition:*** *Surveys in which the respondent communicated that the survey does not apply to their facility and meets at least one of the criteria described below.*	
We largely operated in a blind recruitment strategy in the sense that we had no details about the facilities in the *Compendium* other than the information provided to us. However, the study team accounted for this by incorporating an initial screener question about whether the facility houses pregnant women to determine if a survey about pregnancy and opioid use disorder would apply. If the respondent selected ‘no,’ they were instructed to end the survey [and mail it back for Wave 2]. Of the 226 categorized as not applicable, the majority (*n* = 142) do not house females or pregnant females. We believe the subject may have contributed slightly to lower response rates. Additionally. Since the contact persons in the *Compendium* are jail administrators rather than clinicians, the topic area may not have resonated. The study team used the information respondents provided to group ‘not applicable’ situations into five non-exclusive main categories:	
(1) Don’t house females and/or pregnant females (n = 142)	
(2) No jail at this location (n = 76)	
(3) Don’t operate/run a (the) jail (*n* = 6)	
(4) Short-term holding facility/send elsewhere for medical care (*n* = 13)	
(5) Don’t accept detainees that meet these criteria (pregnant with OUD) (n = 2)	
(6) House detainees in another facility/county (*n* = 38)*	
The surveys classified as ‘not applicable’ yielded valuable information about research involving jails and how they operate. **The following case example highlight inconsistencies in communication and knowing which facilities provide what services.**	
**Case Example #1:** Don’t house females and/or pregnant females.	
Two facilities in the same county were surveyed. Facility A responded requesting the survey be forwarded to Facility B. Facility B responded reporting they don’t house pregnant females.	
**Case Example #2:** Reports of no jail at the surveyed location.	
One respondent communicated to the study team that the county jail closed several years before but recommended we forward the survey to another facility in which they provided the address. When the forwarded survey was received, a different respondent replied with *“We don’t have a jail. The jail is a regional jail.”*	
**Case Example #3:** Reports of we do not run/operate a jail.	
Many facilities responded with reports that they either don’t run the jail or operate a jail which questions how they met the criteria to be included in the *Compendium*.	
[County name] reported they do not run the jail, asked us to direct it to the prison and provided an address—the address is the same address this person responded from.	
**Case Example #4:** Some respondents communicated with the study team to express that the survey was not applicable because they were short-term holding facilities.	
*“When I started into the survey I thought I could answer all the questions about the very small population that my office has incarcerated over the years. But I find myself not knowing the answers because we do not hold females in our facility. I have a 72 h holding facility. All females are transported to other facilities who have the programs in the survey. I am not aware which program or how they address any opioid addiction they might have. Your survey has helped me to investigate further into the jails I utilize on short term and longer term basis. I am sorry I cannot complete your survey, I would rather not answer questions I do not know the answer too.”* *“I am not sure our Detention Facility applies for many of these questions since we are not a long-term housing facility. We do not provide medical care other than Paramedics when needed. If one of our female inmates is pregnant, and there would be any medical complications, we will transport that inmate to the court holding their warrant (as a county would have medical staffing). If the inmate is here for something local, we would release the inmate to the paramedics who would then transport him/her to the hospital.”*	
**Case Example #5:** Some facilities reported that they would not accept a detainee that was pregnant and had opioid use disorder.	
*“Not available in our facility. We are too small to deal with this problem. Female would be sent to a facility that is equipped.”* *“We don’t accept inmates falling in this category. We are in the infancy stages of talking about a MAT program.”*	
*The study team encountered a number of facilities responding to the survey who indicated they house their detainees in another facility or county (*n* **= 38). Because the survey is technically not applicable to them for this reason, the study team counted them towards the ‘not applicable’ total despite being described in the ‘Location Issues’ section. This, coupled with the responses indicated no jail at said location, questions why those facilities were included in the** ***Compendium*** **and what constitutes a jail, especially for research involving health services.**	
**Location** **Challenges**	
***Definition:*** *Surveys that reveal complexities regarding the physical area of jurisdiction (*i.e. *county), have some physical overlap with other facilities in the Compendium and meet one or more of the criteria outlined below.*	
As previously explained in the section that describes the inclusion and exclusion criteria used when creating the *National Jails Compendium*, both county- and city-level jails were included. We also found a number of regional jails that house detainees from multiple counties. Our study team decided to survey all facilities in the *Compendium* to capture as many institutions of detention as possible which has revealed many challenges when accounting for geographical coverage areas. On the survey, we asked respondents to report their county for tracking purposes. We then followed by asking respondents to report if they oversee jails in more than one county, and if yes, identify those counties. We took common themes from returned surveys (*n* = 141) that exhibited location challenges and organized their responses into the following categories:	
(1) City jail located within a county that was also surveyed (*n* = 19)	
(2) Regional jail that covers multiple counties (n = 13)	
(3) Facility reported the same county as another surveyed facility (*n* = 24)	
(4) Facility (not identified as a regional jail) lists multiple counties (n = 33)	
(5) House detainees in another facility/county (*n* = 28)	
(6) Survey response accounts for more than one facility [different physical address but same contact person] (n = 25)	
**Case** **Example** **#1: Responses from city jails that are located in counties where a county facility was also surveyed**.	
Many respondents from city jails indicated they are short-term holding facilities or they don’t house pregnant females, information that could not be gleaned about the jails through publically available information. In other instances, they responded with complete surveys noting their county affiliation. We did not receive any correspondence from city facility that indicated any overlap or connection with the county facility and vice versa.	
**Case** **Example** **#2: Facilities that are recognized as** **Regional jails** **[based on the institution name]** **and hold detainees from multiple counties**.	
A number of regional jail facilities were included in the *Compendium*. Additionally, a number of jails in the compendium reported that they contract with a regional jail or house their detainees at a regional facility. *“[County name] does not currently have a jail. We contract with [regional jail name].”* *“We are a law enforcement agency with no jail. We are partners with 6 other Sheriff’s Offices to form a regional jail, [regional jail name].”* *“[County name] is part of a multi-county jail located in [City, State]. I do not oversee the jail, so I cannot give you the information that you are asking for. Please send a survey to the [facility name] at [facility address].”* *“This agency does not have a jail. We use [regional jail name] located in [City, State].”* *“[County name] does not have jail. All inmates are sent to [regional jail name] in [city, state].”* *“[County name] does not operate a jail. We are a part of the [regional jail name] and it is operated by the Jail Authority—not the Sheriff of [county name].”*	
**Case** **Example** **#3: Facilities with a different physical address and contact person but reported the same county of at least one other facility in the** **Compendium**.	
The study team intentionally cross-referenced county reporting when respondents submitted surveys to flag responses whose county overlapped with other facilities. This revealed a number of surveys who reported overseeing jails within the same county. Those surveys will be further evaluated for similarities in services provided.	
**Case** **Example** **#4: Respondents who reported overseeing jails in multiple counties,** **but are not identified as a regional jail**.	
Similarly, some facilities reported overseeing jails in multiple counties. Those surveys were also grouped so the study team could get a better understanding of their geographic coverage area and how it overlaps with other facilities in the *Compendium*.	
**Case** **Example** **#5: Respondents who indicated they house their inmates in another facility/county**.	
We received a number of notes from facilities reporting they house their detainees in other counties. In some instances, they identified those other facilities and/or counties. In almost every case, those identified facilities were also a part of the *Compendium*. *“We do not have a jail in our facility. All of our arrests go to the [jail name].”* *“I just wanted to touch base with you and let you know that we do not have an incarceration facility in our county. We house all of our inmates at [detention facility name] in [city, state].”* *“I would love to help you out with this but we don’t have a jail. We contract with [jail name] and [jail name].”* *“The [county] Sheriff’s Office doesn’t house prisoners, we take them to the [detention facility name].”* *“[County name] no longer has a correctional facility, closed in 2017. Combined facility with [county name].”* *“All inmates for [county name] [city, state] are housed at [detention facility name and address]. They would have this information.”* *“We no longer (as of 2004) run a city jail. [Jail name] now houses our prisoners. Please contact me if you have questions.”*	
**Case** **Example** **#6: Survey response accounts for more than one facility with a different physical address but had the same contact person listed in the** **Compendium**.	
The *Compendium* listed multiple facilities with the same contact person. In electronic recruitment, that person was only emailed once, but in Wave 2 recruitment, a paper survey was sent to every address to maximize the chances of return. In some cases, those surveys were filled out multiple times. Those respondents are from the same county, have a different facility address but same contact person. However, the person filling out the survey could vary. One jail with three different detention facilities (different addresses) but the same contact person responded to all three Wave 2 survey attempts. At least 2 different people filled out the surveys. The surveys were filled out differently and communicate different provision of health care services and treatment protocols. **Physical area of jurisdiction is essential in mass data collection and should be accounted for when surveying institutions that operate and are defined differently from state to state and even within states. Physical jurisdiction and operational oversight are important with research involving jails particularly because jail officials are elected. These factors influence how we are categorizing responses and the availability of health services. They also demonstrate the complexities of jail research and the usefulness of the** ***Compendium.***	

## Discussion

### Value of the compendium

To our knowledge, this is the largest research survey to date of health care services in jails across the U.S. While the Centers for Disease Control and Prevention (CDC) conducted a survey of state prisons regarding the availability of various health care services, no such similar comprehensive study exists of jails (Chari et al., 2016). Our study involving the availability of MOUD for pregnant people speaks to the utility and challenges of using the *Compendium* for health-related research.

The processes of compiling contact information about jails, soliciting health care services information, and processing (non-)responses provide useful information for future researchers. Moreover, research with the *Compendium* illuminates core issues about accountability for these public institutions.

Given the vast influence of the U.S. jails system, a national jails datasheet has immense research value. Publicly available information about jails is inconsistent and difficult to find. The goals of the *Compendium* are therefore twofold. First, the *Compendium* addresses the knowledge gap about jails, aiming to improve information on jails. Second, the *Compendium* aims to facilitate U.S. jail-based research by reducing the work required by researchers in locating jails, in addition to decreasing the chances of missing facilities. By creating the *Compendium*, we hope research in jails becomes less encumbered. Additionally, we hope the *Compendium* sheds light on the less frequently researched, but equally important, facilities such as city jails and smaller county jails, as well as increases knowledge about the overall structure and operations of U.S. jails. Given these goals, the *Compendium* is available upon request from Stephen Amos at the NIC/BOP (Stephen.Amos@usdoj.gov). It is our hope that the *Compendium* will facilitate future jail-based research and in turn provide valuable feedback on the information contained within the *Compendium*.

Since the *Compendium* aims at facilitating jail-based research, it is imperative to clearly define what constitutes a jail. The main criteria for inclusion in the *Compendium* are for the jail to be open, active, and house adult populations. The *Compendium* excluded Federal prisons, tribal jails, juvenile facilities, and facilities with only holding cells, and researchers should consider the effect these exclusions have on their study design. Additionally, researchers should be mindful of jail housing structures that distinguish between different populations in different buildings versus different populations on different floors of the same building. These variations could mean different staff and therefore different organizational cultures and relationships, which may provide residents with different experiences.

Aside from the above-mentioned considerations, facility capabilities are an important part of what constitutes a jail. “Length of stay” is particularly important, because it may dictate the availability of services and therefore the scope of future research. Our inclusion of longer-term facilities broadens research possibilities. Facility size is irrelevant for inclusion in the *Compendium,* since smaller and larger facilities often function similarly. Therefore, smaller facilities might be able to shed light on lesser-studied aspects of jails.

In addition to our study, the National Institute of Justice offers guidelines for conducting meaningful research in jails (Chakraborty, 2019), raising issues that complement the lessons we learned in compiling and implementing the *Compendium*. Future researchers can learn from this resource in crafting research questions and designing methods, paired with lessons presented here.

### Information accuracy

One of the challenges of creating a *Compendium* was establishing a starting point. This is in part due to a lack of cohesive information on state websites. In states with Sheriff’s Association websites and published jail information, websites were rarely complete or up to date. Because critical information was often missing, we used other websites and outlets to identify missing variables which reinforces the need for availability of basic information regarding the U. S jail system.

This lack of research on the functioning of jails has implications for facilities themselves, as well as for the research conducted on incarceration more broadly. Given the inaccuracy and sometimes non-existence of information on local jail practices, the total experience of residents in jails may be significantly obscured. This means that research on incarceration may be missing out on significant variables impacting resident behavior and outcomes. Community relationships may also be obscured, which may impact how jails function on a day-to-day basis. Additionally, more transparency and research in jails can benefit jails as well. Different facilities are constantly developing innovative ways of handling day-to-day operations, which can be beneficial to other jails in different areas of the U.S. More transparency in how jails function may eventually save money, improve resident and staff conditions within facilities, and overall improve the way jails function.

Creating a more transparent environment is no easy task. State and local governments could aid in improving transparency and the research process by improving upon processes already in existence. For example, Sheriff’s Associations are already centralized, local agencies. Since jails are often connected to sheriff departments, capitalizing upon these connections by maintaining record of local practices and facilities could improve upon the current transparency. We hope that the issues our team encountered in collecting reliable, up to date information brings awareness to local and state governments on the difficulties of contacting people within the jail. As such, it is possible that loved ones have similar issues with contacting these facilities. Improving the ease through which facilities can be contacted and the information that is publicly available may help relationships within the community for facilities and residents simultaneously, and eventually make daily functioning easier for jails themselves as well.

Because out-of-date websites may reflect inaccurate information about facilities, frequent updates to the *Compendium* are imperative. Facilities may have changed leadership, closed, relocated, or changed their housing policies without reflecting these changes on their websites. Discovering which facilities have information inaccuracies is difficult until researchers use the *Compendium* and provide feedback.

Our methods for compiling the *Compendium* have some limitations. Because the project was vast and substantial, it is possible the team missed qualifying jails. While we tried to be comprehensive, not all websites were informative, and we therefore resorted to a litany of alternate ways of gathering information. These information-gathering strategies do not guarantee accuracy. However, being aware of these limitations, the team cross-referenced all found jails and used multiple checks throughout the process.

### Lessons from returned surveys

Aside from BJS’s periodic census of jails, national-scale research about jails is rare and frequently relies on surveying a representative sample. The inconsistencies and challenges of locating jails has, until the *Compendium*, precluded researchers’ abilities to conduct studies within jails. Despite the Compendium’s rigorous search, we received responses from unidentified jails, due to our snowball sampling and outreach via NSA. Jails open and close, change leadership, and may have slipped through the cracks of the *Compendium* search strategy.

Strategic data collection and recruitment processes are needed to not only reach jail facilities, but to also maximize response rates. BJS performs a jail census through field visits, phone calls, and mailing forms, rather than doing so electronically, but this is not practical for health services researchers. The ARRWIP team used an endorsement and logo from an organization jails respect and recognize, the NSA, in recruitment materials, sent pre-survey postcards, invited snowball sampling, and used both electronic and mailed forms. In 1999, a CDC study involving an email survey to 115 jail facilities had a 97% response rate, though this was calculated by county (not facility) and occurred at a time when email correspondence was still infrequent (Parece, Herrera, Voigt, Middlekauff, & Irwin, 1999). We hypothesized that electronic recruitment would yield a higher response rate than paper recruitment because it required less effort from the respondent and reduced chances of lost surveys. However, the bulk of the responses came from paper surveys (*n* = 769) compared to electronic submissions (*n* = 403). This overall low response rate of 10% to the electronic survey suggests future jail surveys might use paper surveys, but this comes with a warning that our paper recruitment effort was intensive and required more people, time, and money. Paper responses also increased the chances of data quality concerns (i.e., respondents completing the survey incorrectly). Despite these hurdles, the responses we received via paper surveys were invaluable.

Mailed-back surveys that we deemed “not applicable” for our survey about pregnant women with OUD provided telling information about jails and stressed the need for a regularly updated national jails database. Many responses came with handwritten notes explaining: (1) there is no jail at that location; (2) the jail is a short-term holding facility, or (3) detainees are housed in another facility and/or county. These responses shed light on what constitutes a jail, what services jails may or may not provide, where people are housed, and whether the jail actually exists.

Some of the inner workings of jails were illuminated through duplicate survey responses. Duplicate surveys were sometimes submitted by the same person, but most often completed by two different jail leaders and communicated different policies and practices at the same facility. Miscommunication between leadership is problematic, but the reporting of different medical policies and practices by people at the same jail is a broader issue that suggests jail leadership may not know what services they provide. The complexities of jails’ operations alone warrant more research involving jails and the necessity of a *National Jails Compendium*.

## Conclusions

Jails are public institutions, funded by taxpayers who have a reasonable expectation for transparency. Communities surrounding jails should have means of contacting facilities with questions or concerns, as should individuals attempting to find loved ones. People enter and leave jails every day; thus, we must recognize jails as integrated within, not isolated from, community health and other systems. The extensive efforts our team employed to enumerate and locate contact jails reflects a lack of transparency. While the Freedom of Information Act provides a mechanism for any person to request a record from a public institution (FOIA, n.d.), this process is cumbersome and does not guarantee a response.

Research and transparency about jails is not simply about cataloging information; rather, it has implications for daily operations of jails and for humane, safe treatment of incarcerated people. Jails may be apprehensive of participation in research, but research informing best practices is beneficial. Disseminating research findings can help jails across the country learn from other jails, improve their health care services, and better serve the community. Given how many millions of people pass through U.S. jails every year, that the U.S. carceral system disproportionately confines people of color, and that these are public institutions, the comprehensive and accurate study of this vast network of jails is a matter of equity and justice. The *Compendium* is a critical first step in such jail-focused work.

## Data Availability

The data that support the findings of this study are available from the National Institute of Corrections but restrictions apply to the availability of these data, which were used under license for the current study, and so are not publicly available. Data are however available from the authors upon reasonable request and with permission of the National Institute of Corrections.
